# A phenomenological model for structural phase transitions in incommensurate alkane/urea inclusion compounds

**DOI:** 10.1098/rsos.180058

**Published:** 2018-06-13

**Authors:** Michel Couzi, François Guillaume, Kenneth D. M. Harris

**Affiliations:** 1 Université de Bordeaux, CNRS, ISM UMR 5255, 351 cours de la Libération, 33405 Talence Cedex, France; 2 School of Chemistry, Cardiff University, Park Place, Cardiff CF10 3AT, UK

**Keywords:** incommensurate composite materials, solid-state phase transitions, superspace groups, urea inclusion compounds

## Abstract

*n*-Alkane/urea inclusion compounds are crystalline materials in which *n*-alkane ‘guest’ molecules are located within parallel one-dimensional ‘host’ tunnels formed by a helical hydrogen-bonded arrangement of urea molecules. The periodic repeat distance of the guest molecules along the host tunnels is incommensurate with the periodic repeat distance of the host substructure. The structural properties of the high-temperature phase of these materials (phase I), which exist at ambient temperature, are described by a (3 + 1)-dimensional superspace. Recent publications have suggested that, in the prototypical incommensurate composite systems, *n*-nonadecane/urea and *n*-hexadecane/urea, two low-temperature phases II and ‘III’ exist and that one or both of these phases are described by a (3 + 2)-dimensional superspace. We present a phenomenological model based on symmetry considerations and developed in the frame of a pseudo-spin–phonon coupling mechanism, which accounts for the mechanisms responsible for the I ↔ II ↔ ‘III’ phase sequence. With reference to published experimental data, we demonstrate that, in all phases of these incommensurate materials, the structural properties are described by (3 + 1)-dimensional superspace groups. Around the temperature of the II ↔ ‘III’ transition, the macroscopic properties of the material are not actually associated with a phase transition, but instead represent a ‘crossover’ between two regimes involving different couplings between relevant order parameters.

## 1. Introduction

Many nanoporous organic crystalline materials can be described as host–guest composite systems constructed from two interpenetrated substructures. Urea inclusion compounds [1] are a widely studied family of host–guest composite materials in which ‘guest’ molecules are located within parallel one-dimensional ‘host’ tunnels formed by a helical hydrogen-bonded arrangement of urea molecules. The urea host structure with empty tunnels is not stable, and the urea tunnel structure exists only when the tunnels are filled by a dense packing of guest molecules. Suitable guest molecules are linear long-chain alkane-based molecules, for which the molecular diameter is similar to the diameter of the urea host tunnels (approx. 5.25 Å).

**Figure 1.**
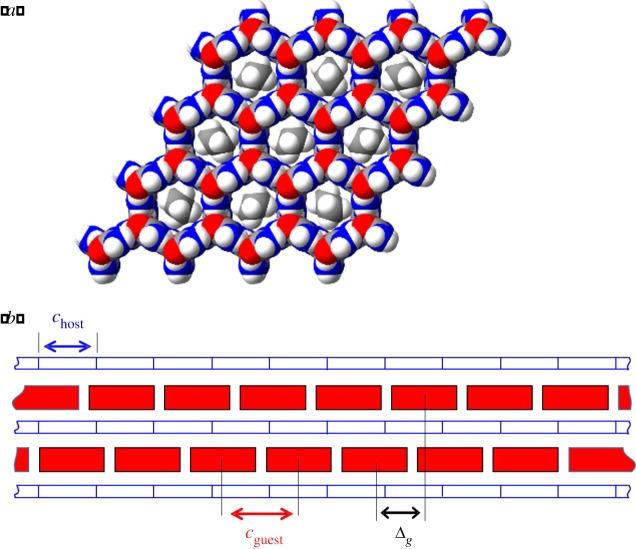
(*a*) Structure of an *n-*alkane/urea inclusion compound at ambient temperature viewed along the tunnel axis. (*b*) Schematic representation of a tunnel inclusion compound viewed perpendicular to the tunnel axis, showing guest molecules (red) arranged along the host tunnel structure (blue). The periodic repeat distances of the host and guest substructures along the tunnel axis are denoted as *c*_host_ and *c*_guest_, respectively. The offset, along the tunnel axis, between the positions of guest molecules in adjacent tunnels is denoted as Δ_g_.

Most urea inclusion compounds containing *n*-alkane [CH_3_(CH_2_)*_m_*CH_3_] guest molecules are incommensurate materials [1–6], with stoichiometry defined by a misfit parameter *γ* = *c*_host_*/c*_guest_, where *c*_host_ and *c*_guest_ are the periodic repeat distances, along the tunnel direction, of the host and guest substructures, respectively [2] (figure 1). The periodicity (*c*_host_) of the host substructure along the tunnel depends on the pitch of the helical arrangement of urea molecules, with *c*_host_ ≈ 11.02 Å for *n*-alkane/urea inclusion compounds at ambient pressure [7,8]. The periodicity (*c*_guest_) of the guest substructure along the tunnel axis depends on the length of the *n-*alkane guest molecule in the extended *all-trans* conformation required to fit inside the urea tunnel structure (for reasons discussed elsewhere [9–11], the value of *c*_guest_ is typically about 0.5 Å shorter than the van der Waals length of the guest molecule). At ambient pressure, the value of the misfit parameter is *γ* = *c*_host_/*c*_guest_ = 0.418 for the *n-*nonadecane/urea (*m* = 17) inclusion compound [6] and *γ* = 0.486 for the *n*-hexadecane/urea (*m* = 14) inclusion compound [12]. Clearly, the different values of *γ* arise from the different lengths of the *n*-nonadecane and *n*-hexadecane guest molecules, which give rise to different values of *c*_guest_.

The basic structure of the urea host substructure is hexagonal with space group *P*6_1_22 (or *P*6_5_22), arising from the helical hydrogen-bonded assembly of urea molecules that forms linear, non-intersecting tunnels parallel to the hexagonal **c**-axis [4,7,8] (figure 1). The composite crystal has sixfold symmetry as a consequence of the fact that the *n-*alkane guest molecules are distributed statistically in at least six energetically equivalent orientations [13,14]. For cases in which the *n*-alkane guest molecules exhibit three-dimensional ordering (i.e. with inter-tunnel ordering of the guest molecules in addition to ordering of the guest molecules along the tunnel axis), another important structural parameter [4] is the offset (denoted by Δ*_g_*), along the tunnel direction, between the positions of guest molecules in adjacent tunnels (Δ*_g_* is defined in figure 1). For most *n*-alkane/urea inclusion compounds [3], the value of the offset is Δ*_g_* = 0, and in this case, the basic guest structure is described [4,15] by space group *P*622. In this situation, the overall symmetry of these host–guest composite materials is described [4] by the (3 + 1)-dimensional superspace group *P*6_1_22(00*γ*), with four integer indices (*h*, *k*, *l*, *m*) required to index all observed reflections in the diffraction pattern:

1.1 $${\text{Q}}_{hklm}=h{\text{a}}^{*}+k\;{\text{b}}^{*}+l\;{\text{c}}_{\mathrm{host}}^{*}+m\;{\text{c}}_{\mathrm{guest}}^{*}.$$

The (*h*, *k*, 0, 0) reflections are main reflections that are common to both the host and guest substructures, the (*h*, *k*, *l*, 0) reflections are main reflections of the host substructure (and also contain information on incommensurate modulations within the guest substructure), the (*h*, *k*, 0, *m*) reflections are main reflections of the guest substructure (and also contain information on incommensurate modulations within the host substructure) and the (*h*, *k*, *l*, *m*) reflections with *l *≠ 0 and *m *≠ 0 are satellite reflections [4–6,8].

**Figure 2.**
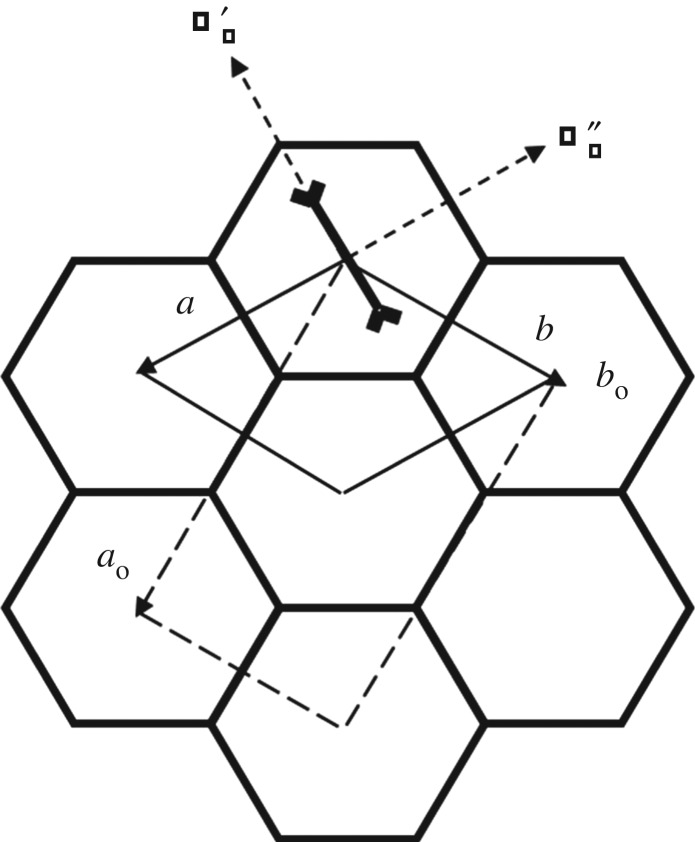
Schematic representation of the hexagonal (*a*, *b*) and orthorhombic (*a*_o_, *b*_o_) unit cells in *n*-alkane/urea inclusion compounds viewed along the tunnel axis (**c**-axis). The thick solid lines represent the projection of the walls of the urea host tunnels onto the *ab-*plane. In the tunnel at the top, an *n*-alkane guest molecule is included (again in projection) to indicate the preferred orientation of the guest molecules relative to the host tunnel.

A wide range of techniques have been used to explore structural phase transitions in *n*-alkane/urea inclusion compounds [1,13,14,16–25]. Early studies reported that these materials undergo a single phase transition at a temperature *T*_c1_ below ambient temperature [20,26,27], with the phase transition temperature depending on the identity (chain length) of the *n*-alkane guest molecule. This phase transition is a ferroelastic transition from the hexagonal phase I above *T*_c1_ to an orthorhombic phase II below *T*_c1_. In the low-temperature phase, the space group of the basic host structure is *P*2_1_2_1_2_1_ (note that *P*2_1_2_1_2_1_ is a subgroup of *P*6_1_22). In the low-temperature phase, a ‘herringbone’ antiferro ordering of the orientations of the guest molecules (projected onto the plane perpendicular to the tunnel axis) exists between adjacent tunnels [14,16,17]. It was later suggested [22] that this phase transition is governed by an antiferro ordering of the *n*-alkane guest molecules affecting both the host shearing and the guest orientations. A theoretical analysis [4] described the phase transition within the framework of a (3 + 1)-dimensional superspace group description of all possible group–subgroup-related host and guest substructures. It followed that phase I is described by superspace group *P*6_1_22(00*γ*) and phase II is described by superspace group *P*2_1_2_1_2_1_(00*γ*). In both phases I and II, the host and guest substructures have the same periodicities in the *ab-*plane (i.e. the plane perpendicular to the tunnel axis), and the misfit parameter *γ* along the tunnel axis (**c**-axis) is the same in phases I and II. In the *ab-*plane, phase II is described by an orthorhombic unit cell which is very similar to the orthohexagonal description of the hexagonal unit cell of phase I but with no *C*-centring. Thus, for phase II, the orthorhombic unit cell is primitive and the lattice parameters *a*_o_ and *b*_o_ are approximately related by $a_{o}\approx b_{o}\sqrt{3}$ (figure 2).

In this paper, we focus on the low-temperature phase transitions (at ambient pressure) in *n*-nonadecane/urea and *n*-hexadecane/urea, as they are the most extensively studied members of the family of incommensurate *n*-alkane/urea inclusion compounds and are regarded as prototypical incommensurate systems.

For *n-*nonadecane/urea, the I ↔ II phase transition occurs at *T*_c1_ ≈ 157 K (on cooling). However, the ‘classical’ description of this phase transition, as discussed above, was questioned recently [28], together with the report of another phase transition at a lower temperature *T*_c2_ ≈ 140 K (on cooling), corresponding to a weak thermal event in DSC data [29]. The ‘new phase’ below *T*_c2_ was denoted as ‘phase III’ [28]. Because the (3, 2, 0, 0) reflection (using the orthorhombic setting discussed above), which is a main reflection for both the host substructure and the guest substructure, was *not* observed in phase II below *T*_c1_, these authors [28] concluded that the orthorhombic unit cell is *C*-centred (i.e. suggesting that the *C*-centre is ‘preserved’ from the orthohexagonal description of the hexagonal phase I), while they concluded that the orthorhombic unit cell of ‘phase III’ below *T*_c2_ is not *C*-centred. Furthermore, these authors also suggested [30] that an additional modulation with period *c_i_* exists along the tunnel axis in both phases II and ‘III’, leading to a second incommensurate misfit parameter *δ* = *c*_host_/*c_i_* in coexistence with the misfit parameter *γ* = *c*_host_/*c*_guest_ along the same direction. Within this interpretation, five integer indices (*h*, *k*, *l*, *m*, *n*) are required to index each reflection in the diffraction pattern:

1.2 $${\text{Q}}_{hklmn}=h\;{\text{a}}^{*}+k\;{\text{b}}^{*}+l\;{\text{c}}_{\mathrm{host}}^{*}+m\;{\text{c}}_{\mathrm{guest}}^{*}+n\;{\text{c}}_{i}^{*}.$$

The indices *m* and *n* relate to the misfit parameters *γ* and *δ*, respectively, with ${\text{c}}_{\mathrm{guest}}^{*}=\gamma {\text{c}}_{\mathrm{host}}^{*}$ and ${\text{c}}_{i}^{*}={\text{a}}^{*}\pm \delta {\text{c}}_{\mathrm{host}}^{*}$, where a* is the reciprocal lattice vector corresponding to the orthorhombic *a*_o_ lattice vector in real space (figure 2). The experimental values reported in [30] for *n-*nonadecane/urea are *γ* = 0.418 and *δ* = 0.090. Space group *C*222_1_(00*γ*)(10*δ*) was assigned [30] to phase II and space group *P*2_1_2_1_2_1_(00*γ*)(00*δ*) was assigned [30] to ‘phase III’.

A similar I ↔ II ↔ ‘III’ sequence of phase transitions has been reported [12] for *n*-hexadecane/urea, with the phase transition temperatures (on cooling) *T*_c1_ ≈ 150 K and *T*_c2_ ≈ 125 K. However, in this case, the symmetry of phase II was described by the (3 + 1)-dimensional superspace group *P*2_1_2_1_2_1_(00*γ*) and the symmetry of ‘phase III’ was described by the (3 + 2)-dimensional superspace group *P*2_1_2_1_2_1_(00*γ*)(00*δ*), and the temperature-independent misfit parameters were reported to be *γ* = 0.486 and (for ‘phase III’) *δ* = 0.058.

However, a very recent paper [31] (based on a high-resolution synchrotron single-crystal X-ray diffraction study of *n*-nonadecane/urea at ambient pressure) refuted the recently reported [28,30] interpretations (summarized above) of the structural properties of *n*-nonadecane/urea and instead demonstrated conclusively that the symmetries of both phases II and ‘III’ can be described by (3 + 1)-dimensional superspace groups, which provide a complete description of the structural properties of these phases. In particular, the misfit parameters *γ* and *δ* in the (3 + 2)-dimensional superspace group description are not independent, as the following relation was proved [31] to exist (within experimental accuracy):

1.3 $$\delta {\text{c}}_{\mathrm{host}}^{*}=-2\;{\text{c}}_{\mathrm{host}}^{*}+5\;{\text{c}}_{\mathrm{guest}}^{*},$$

so that *δ* = –2 + 5*γ*. Correspondingly, the indices (*l*, *m*, *n*) in the (3 + 2)-dimensional superspace group description [30,32,33] are also not independent. On this basis, a (3 + 1)-dimensional superspace group can be defined with only four independent indices (*h*, *k*, *l′*, *m′*), where *l′* and *m′* are related to the indices *l*, *m* and *n* in the (3 + 2)-dimensional superspace description (equation (1.2)) by the following equation:

1.4 $$l^{\prime }=l-2n\;\text{and}\;m^{\prime }=m+5n.$$

It follows that both phases II and ‘III’ of *n*-nonadecane/urea are correctly described by the same (3 + 1)-dimensional superspace group *P*2_1_2_1_2_1_(00*γ*).

Similarly, the symmetry of ‘phase III’ of *n*-hexadecane/urea was shown [31] to be correctly described by a (3 + 1)-dimensional superspace group rather than the (3 + 2)-dimensional superspace group suggested previously [12], as the following relation was proved [31] to exist (within experimental accuracy): *δ* = 2 – 4*γ*.

A comment [34] has been published in response to paper [31] refuting some of our interpretations, but admitting explicitly the existence of the relationships between the two misfit parameters *γ* and *δ* for *n*-nonadecane/urea and *n*-hexadecane/urea quoted above and in paper [31]. Given that the (3 + 2)-dimensional superspace groups proposed previously [12,28,30] for *n*-nonadecane/urea and *n*-hexadecane/urea have been shown [31] to be incorrect, some general observations can be made based on the true degrees of freedom in these systems. Clearly, the orthorhombic distortion is achieved by orientational ordering of the guest molecules about the tunnel axis, coupled with the elastic properties of the host substructure [22]. By contrast, the translational motions of the guest molecules along the tunnel axis do not interact strongly with the host substructure, as evidenced by the molecular transport properties of these materials [35–38]. However, interactions between *n-*alkane guest molecules in adjacent tunnels play a role in the three-dimensional ordering of the guest molecules in the composite host–guest structure, evidenced by the fact that a well-defined value of Δ*_g_* (defined above) is observed for *n*-alkane/urea inclusion compounds (for most *n*-alkane guest molecules, Δ*_g_* = 0, but values of Δ*_g_ *≠* *0 have been reported [32,39] for the *n*-heptane/urea and *n*-dodecane/urea inclusion compounds). Furthermore, an important feature of incommensurate *n*-alkane/urea inclusion compounds is that the misfit parameter *γ* is independent of temperature [12,30].

Within the context of these observations, this paper presents a phenomenological model that accounts for the mechanisms responsible for the I ↔ II ↔ ‘III’ sequence of phase transitions in the *n*-nonadecane/urea and *n*-hexadecane/urea inclusion compounds. Our model is based on symmetry considerations and is developed within the frame of a pseudo-spin–phonon coupling mechanism, as suggested previously [22]. In particular, we show that this model is sufficient to explain the very low intensity (which others [28] may have interpreted as zero intensity) of the (*h*, *k*, 0, 0) reflections with *h + k* odd in phase II of *n*-nonadecane/urea. Moreover, we demonstrate that the differences between phases II and ‘III’ are explained by different couplings that exist between the relevant pseudo-spin variables and the translational motions of the *n*-alkane guest molecules.

## 2. Symmetry properties of *n-*nonadecane/urea and *n-*hexadecane/urea

### 2.1. Group to subgroup relations

In phase I of *n*-nonadecane/urea and *n*-hexadecane/urea, the space group of the basic host structure is *P*6_1_22 and the space group of the basic guest structure is *P*622. Thus, the superspace group based on the host sublattice is *P*6_1_22(00*γ*) with lattice parameters (*a*, *b*, *c*_host_). In both low-temperature phases II and ‘III’, the orthorhombic unit cell is similar to the orthohexagonal description of the hexagonal unit cell of phase I, but with no *C*-centring (figure 2). Thus, we deduce that the transitions to phases II and ‘III’ are related to a lattice instability occurring at a zone boundary point *M*(½, 0, 0), which is a reciprocal point common to both host and guest sublattices in the hexagonal Brillouin zone [40]. This point *M* is replaced at the zone centre (Γ point) in the low-temperature phases II and ‘III’. The wavevector point group at point *M* is 222 (*D*_2_) and the corresponding irreducible representations, denoted as *M*_1_/*A*, *M*_2_/*B*_1_, *M*_3_/*B*_2_ and *M*_4_/*B*_3_, are three-dimensional as there are three arms in the star of this wavevector [40]. Thus, the order parameter associated with each representation has three components *q_i_* (*i* = 1, 2, 3) relative to the three arms of the star of the wavevector. However, in the present case, we only need to consider solutions that belong to the subspaces for which *q_j_* = *Q *≠* *0 for only one of the indices *j* (with *q_i_* = 0 for *i *≠* j*), as only one arm of the star of point *M* is involved in the phase transition [40]. It follows that each equivalent point *M*_1_(½, 0, 0), *M*_2_(0, ½, 0) and *M*_3_(−½, ½, 0) generates a ferroelastic domain, and the three domains are related to each other by the threefold axis collinear with the **c**-axis (tunnel axis) in phase I, which is lost in the orthorhombic low-temperature phase. In fact, an additional splitting of each domain (by about 2° at 90 K) is observed, due to the energy cost of domain walls, resulting in six orthorhombic domains [19].

**Figure 3.**
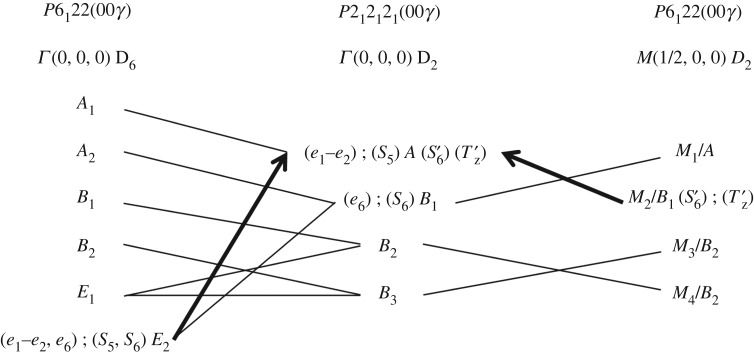
The compatibility relations between the irreducible representations of superspace group *P*6_1_22(00*γ*) at the zone centre point Γ(0, 0, 0) (left column) and at the zone boundary point *M*(½, 0, 0) (right column) with those at the zone centre point Γ(0, 0, 0) of superspace group *P*2_1_2_1_2_1_(00*γ*) (middle column).

Table 1 reports the character table of the critical wavevector group at point *M*(½, 0, 0) corresponding to the subspace *q*_1_ (only the generating symmetry elements are shown). The subgroups induced by each irreducible representation for both the host and guest sublattices [41] and their representation in (3 + 1)-dimensional superspace groups [4] are also shown. It turns out that superspace group *P*2_1_2_1_2_1_(00*γ*) is solely induced by the *M*_2_/*B*_1_ irreducible representation, which thus defines the symmetry of the primary order parameter of the phase transition (i.e. all symmetry properties of the low-temperature phase are determined by this order parameter, provided the misfit parameter *γ* remains constant). In figure 3, we report the compatibility relations that exist between the irreducible representations of space group *P*6_1_22 and those of the zone centre in the *P*2_1_2_1_2_1_ subgroup, including the irreducible representations at point *M* (table 1), as well as those of the Γ point of phase I which remains as a zone centre point in the *P*2_1_2_1_2_1_ subgroup. It transpires that the identity representation *A* of the *P*2_1_2_1_2_1_ subgroup (which necessarily represents the symmetry of the order parameters in the distorted low-temperature phase) is compatible not only with *M*_2_/*B*_1_ but also compatible with the *E*_2_ representation at the zone centre of *P*6_1_22 (figure 3). Such associated order parameters are generally considered as secondary order parameters [42]. Indeed, we note that, when considered alone, the zone centre *E*_2_ representation induces the *C*-centred *C*222_1_ space group, of which *P*2_1_2_1_2_1_ is a subgroup [41]. Such a transition would necessarily be a first-order transition, as the symmetrized third power [*E*_2_]^3^ = *A*_1_ + *A*_2_ + *E*_2_ contains the totally symmetric representation, allowing the presence of a third-order invariant in the Landau free-energy development.

**Table 1.** Character table [40] of the critical wavevector group at point M(½, 0, 0) (only generating symmetry elements are shown) together with the subgroups induced by each irreducible representation for both the host and guest substructures [41] and their description in (3 + 1)-dimensional superspace groups on the basis of the host sublattice [4].

irreducible representation	{*C*_2*z*_|00½}_host_ {*C*_2*z*_|000}_guest_	{$C_{21}^{\prime \prime }$|00½}_host_ {$C_{21}^{\prime \prime }$|000}_guest_	{*E*|100}_host_ {*E*|100}_guest_	induced host subgroup	induced guest subgroup	induced superspace group
*M*_1_/*A*	1	1	−1	*P*222_1_	*P*222	*P*222_1_(00*γ*)
*M*_2_/*B*_1_	1	−1	−1	*P*2_1_2_1_2_1_	*P*2_1_2_1_2	*P*2_1_2_1_2_1_(00*γ*)
*M*_3_/*B*_2_	−1	−1	−1	*P*2_1_22_1_	*P*2_1_22	*P*2_1_22_1_(00*γ*)
*M*_4_/*B*_3_	−1	1	−1	*P*22_1_2_1_	*P*22_1_2	*P*22_1_2_1_(00*γ*)

### 2.2. Pseudo-spins (reorientations)

Single-crystal ^2^H NMR studies [14] of *n*-nonadecane/urea have shown that the *n*-nonadecane guest molecules adopt preferred orientations in both the hexagonal and orthorhombic phases, such that the projection of the plane of the carbon ‘skeleton’ onto the *ab-*plane points towards opposite corners of the hexagonal projection of the host tunnel (figure 2). For *n*-alkanes with *odd* chain length (e.g. *n*-nonadecane), the point symmetry is mm2 (*C*_2*v*_), and the *C*_2_ axis of the *n*-alkane guest molecule coincides with one of the $C_{2}^{\prime }$ axes of the hexagonal host structure (figure 2). For *n*-alkanes with *even* chain length (e.g. *n*-hexadecane), the point symmetry is 2/m (*C*_2*h*_), and the *C*_2_ axis of the *n*-alkane guest molecule coincides with one of the $C_{2}^{\prime \prime }$ axes of the hexagonal host structure (figure 2).

**Figure 4.**
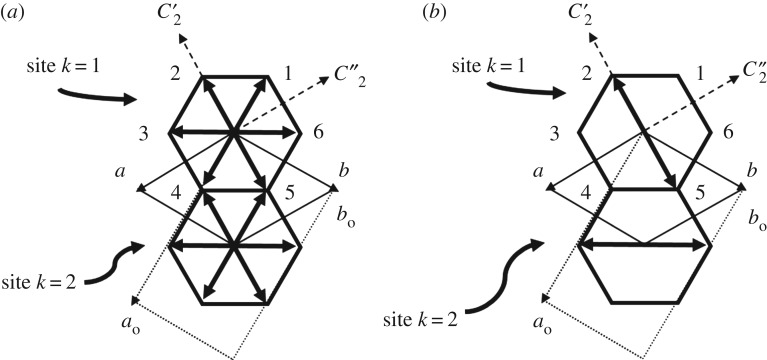
Schematic representation, viewed along the tunnel axis, of the orientational properties of the guest molecules (symbolized by arrows) in *n*-alkane/urea inclusion compounds showing: (*a*) orientational disorder among six equi-probable orientations in hexagonal phase I (space group *P*6_1_22) and (*b*) an ordered arrangement of two preferred orientations in the orthorhombic phase II (space group *P*2_1_2_1_2_1_), leading to a herringbone arrangement of the projections of the guest molecules in adjacent tunnels.

We consider a simple Frankel model of jump reorientations of a rigid *n*-alkane guest molecule with odd chain length about the tunnel axis (**c**-axis), between six energetically equivalent orientations (figure 4) which are related to each other by 60° rotation around the sixfold axis of the tunnel. We use *p_i_* to denote the occupation probability of orientation *i*, with the condition that $\sum_{i=1}^{6}p_{i}=1$ in each phase. In the hexagonal phase I, the statistical sixfold site symmetry of the *n*-alkane guest molecule in each tunnel is achieved when *p_i_* = 1/6 (*i* = 1, 2, … , 6); (figure 4*a*), which describes the time-averaged and space-averaged orientational distribution of the *n*-alkane guest molecules irrespective of the details of the dynamics of the reorientation processes [43–47]. Following the procedure based on the method of projection operators [48], the pseudo-spin variables *s_i_* associated with the orientation of an *n*-alkane guest molecule of odd chain length at a site with 622 (*D*_6_) symmetry in phase I are found to have the following form:

2.1 $$\begin{array}{rl} & A_{1}\;s_{1}=\pm \frac{1}{\sqrt{6}}(p_{1}+p_{2}+p_{3}+p_{4}+p_{5}+p_{6}),\\ & B_{1}\;s_{2}=\pm \frac{1}{\sqrt{6}}(p_{1}-p_{2}+p_{3}-p_{4}+p_{5}-p_{6}\text{) , }\\ & E_{1}\;\left\{ \begin{array}{l} s_{3}=\pm \frac{1}{\sqrt{12}}(2p_{1}+p_{2}-p_{3}-2p_{4}-p_{5}+p_{6})\\ s_{4}=\pm \frac{1}{2}(-p_{2}-p_{3}+p_{5}+p_{6}) \end{array}\right.\\ \;\text{and}\; & E_{2}\;\left\{ \begin{array}{l} s_{5}=\pm \frac{1}{\sqrt{12}}(2p_{1}-p_{2}-p_{3}+2p_{4}-p_{5}-p_{6})\\ s_{6}=\pm \frac{1}{2}(-p_{2}+p_{3}-p_{5}+p_{6}). \end{array}\right. \end{array}$$

The coordinate *s*_1_ belonging to the totally symmetric representation *A*_1_ is merely the identity and hence cannot be a symmetry-breaking order parameter. By contrast, all other coordinates *s_i_* (*i *≠* *1) are symmetry-breaking coordinates and may represent order parameters. We note that, for *n*-alkanes of even chain length, coordinates *s_i_* of the same form are established, except that the coordinate *s*_2_ now belongs to the representation *B*_2_ instead of *B*_1_. This does not change anything in our model, as neither the *B*_1_ representation nor the *B*_2_ representation is involved in the phase transition mechanism, as shown in the following discussion.

Within the orthorhombic unit cell of the low-temperature phase, there are two *n*-alkane guest molecules (labelled *k* = 1 and *k* = 2) and two urea tunnels per unit cell when projected onto the *ab-*plane (figure 4*b*). The positions of the guest molecules with *k* = 1 and *k* = 2, expressed relative to the hexagonal unit cell of the guest substructure in phase I, are as follows (figure 4*b*):

*k* = 1: positions (0, 0, 0), (0, 1, 0), (0, 2, 0), etc.
*k* = 2: positions (1, 0, 0), (1, 1, 0), (1, 2, 0), etc. deduced from the *k* = 1 positions by the translation {*E*|100}.

It follows that the relevant pseudo-spin variables *S_i_* developed on the basis of the site coordinates *s_i_* at the zone centre (i.e. those variables belonging to *E*_2_ symmetry; figure 3) have the following form:

2.2 $$E_{2}\left\{ \begin{array}{l} S_{5}=s_{5}^{1}+s_{5}^{2}\\ S_{6}=s_{6}^{1}+s_{6}^{2} \end{array}\right.,$$

where the superscript (*k* = 1, 2) of the coordinates $s_{i}^{k}$ refers to the labels for the two guest sites specified above. At point *M*(½, 0, 0) of the Brillouin zone, the sole $S_{i}^{\prime }$ pseudo-spin variable with *M*_2_/*B*_1_ symmetry (figure 3) is

2.3 $$M_{2}/B_{1},\;S_{6}^{\prime }=s_{6}^{1}-s_{6}^{2}.$$

Following the arguments developed in §2.1, we establish that the $S_{6}^{\prime }$ pseudo-spin variable with *M*_2_/*B*_1_ symmetry (equation (2.3)) acts as a primary order parameter for the hexagonal-to-orthorhombic phase transition, while *S*_5_ (equation (2.2)) is a secondary order parameter (figure 3). From equations (2.1–2.3), we also establish that the ground state corresponding to the expected ‘herringbone’ arrangement of the guest molecules in the low-temperature phase (see §1) [14,16,17] is achieved under the concomitant actions of *S*_5_ and $S_{6}^{\prime }$ (with *S*_5_* *≠* *0 and $S_{6}^{\prime }\neq 0$), corresponding to

2.4 $$p_{2}^{1}=p_{5}^{1}=p_{3}^{2}=p_{6}^{2}=\frac{1}{2}.$$

As shown in figure 4*b*, this situation does indeed correspond to a ‘herringbone’ arrangement of the *n*-alkane guest molecules in the low-temperature orthorhombic phase. This description also implies that the ground state is disordered, with the *n*-alkane guest molecules occupying two equivalent orientations in each site, related by the {*C*_2*z*_|000} symmetry operation of the orthorhombic *P*2_1_2_1_2 space group of the basic guest structure (table 1), in agreement [4] with orientational disorder of the *n*-alkane guest molecules in superspace group *P*2_1_2_1_2_1_(00*γ*). As a consequence, a ‘frozen’ orientational disorder resulting in a ‘glassy’ crystalline state is expected at very low temperature; indeed, an excess heat capacity has been detected [49] between *T* = 0.1 K and *T* = 0.7 K for *n*-nonadecane/urea, which is well described by a two-level system model and could then be the signature of an orientational ‘glassy’ state due to the guest substructure.

### 2.3. Translations (acoustic modes)

For the urea host substructure, the pure translational motions close to the Brillouin zone centre are the acoustic modes. The propagation of these modes depends on the elastic constants of the crystal, and hence are related to the spontaneous strain that takes place at a ferroelastic phase transition (see §3, equation 3.4). In the present case, the elastic tensor components in the hexagonal phase transform as *A*_1_(*e*_1_+*e*_2_, *e*_3_), *E*_1_(*e*_4_, *e*_5_) and *E*_2_(*e*_1_–*e*_2_, *e*_6_) symmetries [50,51]. It follows that the spontaneous strain *e*_s_ associated with the (*e*_1_–*e*_2_) strain tensor component responsible for an orthorhombic distortion is a secondary order parameter together with the *S*_5_ pseudo-spin coordinate (figure 3). The associated elastic constants are (*c*_11_–*c*_12_) = 2 *c*_66_.

For the *n*-alkane guest substructure, the unique pure translational degree of freedom at the zone centre is the ‘sliding mode’, which represents translation of the *n*-alkane guest substructure as a whole relative to the urea host substructure along the tunnel axis. The sliding mode is analogous to the phason mode in modulated incommensurate systems, and hence, it is expected to exhibit very low (but non-zero) activation energy. Indeed, a diffusive or over-damped mode in the range of 0.8 meV that could be related to a sliding mode with a gap has been reported at the zone centre [25,52]. In any case, the sliding mode, which is polarized along the **c**-axis and hence belongs to *A*_2_ symmetry at the zone centre, cannot be related to any order parameter, provided that the misfit parameter *γ* remains constant (figure 3).

In both the host and guest substructures, the longitudinal acoustic (LA) and transverse acoustic (TA) modes at point *M*(½, 0, 0) transform as *M*_4_/*B*_3_ (TA*_x_* polarized along the **a**-axis), *M*_3_/*B*_2_ (LA*_y_* polarized along the **b**-axis) and *M*_2_/*B*_1_ (TA*_z_* polarized along the **c**-axis). The TA*_z_* mode of the guest substructure (denoted as $T_{z}^{\prime }$ in figure 3) is of particular interest as it belongs to the *M*_2_/*B*_1_ representation of the order parameter responsible for the loss of *C*-centring in the orthorhombic low-temperature phase. Furthermore, it corresponds to pure translational motions of the *n*-alkane guest molecules along the tunnel axis, which are alternately out of phase between successive tunnels along the [110] direction of the orthorhombic unit cell (figure 4). This mode can therefore be viewed as a zone boundary sliding mode, probably associated with a low activation energy. ‘Freezing’ of such a motion will result in a static configuration in which the *n*-alkane guest molecules in successive tunnels along the [110] direction are alternately displaced along the **c**-axis by +*δ*_g_/2 or −*δ*_g_/2 from their average positions.

## 3. The pseudo-spin phonon coupling mechanism

On the basis of symmetry properties and a pseudo-spin translation coupling mechanism, we have identified two primary order parameters belonging to the zone boundary *M*_2_/*B*_1_ representation, namely the $T_{z}^{\prime }$ zone boundary sliding mode (denoted as *Q*_1_) and the $S_{6}^{\prime }$ pseudo-spin coordinate (denoted as *Q*_2_). Furthermore, two secondary order parameters belonging to the zone centre *E*_2_ representation have been identified, namely the *S*_5_ pseudo-spin coordinate and the spontaneous strain *e*_s_ corresponding to the component (*e*_1_–*e*_2_) of the strain tensor (figure 3). Different couplings between these variables are allowed by symmetry. In particular, bilinear coupling between *Q*_1_ and *Q*_2_ (which both belong to *M*_2_/*B*_1_ symmetry) of the form *Q*_1_*Q*_2_ is allowed, and bilinear coupling between *S*_5_ and *e*_s_ (which both belong to *E*_2_ symmetry) of the form *S*_5_*e*_s_ is allowed. Furthermore, linear–quadratic coupling terms are allowed between primary and secondary order parameters of the form (*Q*_1_)^2^*S*_5_, (*Q*_1_)^2^*e*_s_, (*Q*_2_)^2^*S*_5_ and (*Q*_2_)^2^*e*_s_.

A complete treatment would consider four competing ‘full’ order parameters *S*_5_, *e*_s_, *Q*_1_ and *Q*_2_, each exhibiting its own temperature dependence and all dependent on each other. Such a complicated situation is unworkable in practice using classical analytical methods, unless some simplifications are adopted. First, we limit our discussion to the subspace of the wavevector group at point *M* corresponding to *q*_1 _≠ 0 with *q*_2_ = *q*_3_ = 0 (see §2.1). In this context, starting from the hexagonal high-temperature phase I (*S*_5_ = *e*_s_ = *Q*_1_ = *Q*_2_ = 0) and provided the misfit parameter *γ* remains unchanged between the high-temperature and low-temperature phases (see §2.1), only two solutions for the low-temperature phases II and ‘III’ are induced by the *M*_2_/*B*_1_ and *E*_2_ representations, specifically the *C*222_1_(00*γ*) superspace group (corresponding to *S*_5 _≠ 0, *e*_s _≠ 0, *Q*_1_ = 0, *Q*_2_ = 0) and the *P*2_1_2_1_2_1_(00*γ*) superspace group (corresponding to *S*_5 _≠ 0, *e*_s _≠ 0, *Q*_1 _≠ 0, *Q*_2 _≠ 0). Furthermore, it has been shown that both phases II and ‘III’ belong to the same *P*2_1_2_1_2_1_(00*γ*) superspace group [31]. As the phase with superspace symmetry *C*222_1_(00*γ*) remains a virtual phase at ambient pressure, we adopt a shortcut by considering that, as a first approximation, *Q*_1_ and *Q*_2_ behave as ‘full’ order parameters (i.e. with their own temperature dependences) and that S_5_ and *e*_s_ with *E*_2_ symmetry behave as ‘classical’ secondary order parameters. We note that the same approach was adopted by Breczewski *et al.* [53] to describe the ferroelastic phase transition in *n*-heptadecane/urea, but they considered only one zone boundary order parameter involved in the transition instead of the two coupled order parameters *Q*_1_ and *Q*_2_ used here.

In the present case, the temperature dependences of *S*_5_ and *e*_s_ are entirely related to those of *Q*_1_ and *Q*_2_ through the coupling terms (*Q*_1_)^2^*S*_5_, (*Q*_1_)^2^*e*_s_, (*Q*_2_)^2^*S*_5_ and (Q_2_)^2^*e*_s_, which prevent the formation of the phase with *C*222_1_(00*γ*) symmetry. Furthermore, we suppose that the order parameter *Q*_1_ first condenses at *T*_c1_, and that *Q*_2_ is related to the thermal anomaly at *T*_c2_ (*T*_c1_ > *T*_c2_). At this stage of our analysis, this choice is arbitrary and could be reversed, as discussed in §4. Accordingly, we express the Landau free-energy potential developed up to sixth order as follows:

3.1 $$\begin{array}{rl} \Delta F & =\frac{1}{2}A_{1}{\left(Q_{1}\right)}^{2}+\frac{1}{4}B_{1}{\left(Q_{1}\right)}^{4}+\frac{1}{6}C_{1}{\left(Q_{1}\right)}^{6}+\frac{1}{2}A_{2}{\left(Q_{2}\right)}^{2}+\frac{1}{4}B_{2}{\left(Q_{2}\right)}^{4}+\frac{1}{6}C_{2}{\left(Q_{2}\right)}^{6}+DQ_{1}Q_{2}\\ & \;+\frac{1}{2}E{\left(S_{5}\right)}^{2}+\frac{1}{2}FS_{5}{\left(Q_{1}\right)}^{2}+\frac{1}{2}c_{11}^{0}({\left(e_{1}\right)}^{2}+{\left(e_{2}\right)}^{2})c_{12}^{0}e_{1}e_{2}+G(e_{1}-e_{2}){\left(Q_{1}\right)}^{2}, \end{array}$$

where $c_{11}^{0}$ and $c_{12}^{0}$ are the ‘bare’ elastic constants [50] such as $(c_{11}^{0}-c_{12}^{0})=2c_{66}^{0}$. For simplicity, we have retained only the bilinear coupling term *Q*_1_*Q*_2_ and the linear–quadratic coupling terms (*Q*_1_)^2^*S*_5_ and (*Q*_1_)^2^*e*_s_. All other allowed couplings between *Q*_2_, *S*_5_ and *e*_s_ (i.e. (*Q*_2_)^2^*S*_5_ and (*Q*_2_)^2^*e*_s_) are neglected because the transition at *T*_c2_ does not produce any detectable anomaly in the temperature dependence of *e*_s_ (see §4). Also, no Lifshitz invariant or gradient terms are considered because the misfit parameter *γ* (i.e. the parameter that describes *all* the incommensurate properties of the *n-*alkane/urea inclusion compounds) remains temperature-independent through the observed phase transitions. As we must account for two thermal anomalies at low temperature, occurring at *T*_c1_ and *T*_c2_, we need to introduce two critical temperatures, which we denote as *T*_1_ (the temperature at which *A*_1_ changes sign) and *T*_2_ (the temperature at which *A*_2_ changes sign):

3.2 $$A_{1}=a_{1}(T-T_{1})\;\text{and}\;A_{2}=a_{2}(T-T_{2}),$$

with *a*_1_ > 0 and *a*_2_ > 0. The spontaneous strain is defined as follows:

3.3 $$e_{s}=(e_{1}-e_{2})=\frac{a_{o}-b_{o}\sqrt{3}}{a_{o}}.$$

The minimization equations $(\partial \Delta F/\partial S_{5})=0$ and $(\partial \Delta F/\partial e_{i})=0$ (*i* = 1, 2) give

3.4 $$S_{5}=-\frac{F}{E}{\left(Q_{1}\right)}^{2}\;\text{and}\;e_{s}=-\frac{2G}{\left(c_{11}^{0}-c_{12}^{0}\right)}{\left(Q_{1}\right)}^{2},$$

so that $e_{s}=((2GE)/(F(c_{11}^{0}-c_{12}^{0})))\frac{}{}S_{5}$.

Thus, *e*_s_ is directly proportional to *S*_5_, as expected. Then, equation (3.1) can be rewritten as follows:

3.5 $$\Delta F=\frac{1}{2}A_{1}{\left(Q_{1}\right)}^{2}+\frac{1}{4}B_{1}^{\prime }{\left(Q_{1}\right)}^{4}+\frac{1}{6}C_{1}{\left(Q_{1}\right)}^{6}+\frac{1}{2}A_{2}{\left(Q_{2}\right)}^{2}+\frac{1}{4}B_{2}{\left(Q_{2}\right)}^{4}+\frac{1}{6}C_{2}{\left(Q_{2}\right)}^{6}+DQ_{1}Q_{2},$$

where

3.6 $$B_{1}^{\prime }=B_{1}-\frac{2{\left(F\right)}^{2}}{E}-\frac{4{\left(G\right)}^{2}}{\left(c_{11}^{0}-c_{12}^{0}\right)}.$$

A similar form of the free-energy expansion has been considered by Salje and coworkers [51,54–56] to describe the phase transition in sodium feldspar. The condition that the system is in thermodynamic equilibrium for any combination of the order parameters is

3.7 $$\frac{\partial \Delta F}{\partial Q_{1}}=\frac{\partial \Delta F}{\partial Q_{2}}=0,$$

which leads to two basic equations that must be satisfied simultaneously:

3.8 $$A_{1}Q_{1}+B_{1}^{\prime }{\left(Q_{1}\right)}^{3}+C_{1}{\left(Q_{1}\right)}^{5}+DQ_{2}=0$$

and

3.9 $$A_{2}Q_{2}+B_{2}{\left(Q_{2}\right)}^{3}+C_{2}{\left(Q_{2}\right)}^{5}+DQ_{1}=0.$$

Only two solutions of equations (3.8) and (3.9) exist, which define two thermodynamic phases:

3.10 $$\text{phase I}:P6_{1}22(00\gamma ):Q_{1}=0,Q_{2}=0$$

and

3.11 $$\text{phase II}:P2_{1}2_{1}2_{1}(00\gamma ):Q_{1}\neq 0,Q_{2}\neq 0.$$

No further phase described by only one order parameter is stable for *D* ≠ 0. However, the uncoupled expression in *Q*_1_ and *Q*_2_ (i.e. with *D* = 0 in equation (3.5)) would produce two successive phase transitions around *T*_1_ and *T*_2_ (equation (3.2)). Hence, for $B_{1}^{\prime }>0$ and *B*_2_ > 0, a second-order phase transition will occur at a temperature *T*_c_ between *T*_1_ and *T*_2_. For $B_{1}^{\prime }<0$ and *B*_2_ > 0, a first-order phase transition (as observed experimentally) will occur at a temperature *T*_c_ = *T*_c1_ with *T*_c_ > *T*_1_ and *T*_c_ > *T*_2_ (note that the signs of $B_{1}^{\prime }$ and *B*_2_ are not imposed by any symmetry constraints). Importantly, the thermal anomaly at *T*_c2_ is no longer associated with a phase transition, but should be viewed instead as a ‘crossover’ point between two regimes [51] involving two-order parameters *Q*_1_ and *Q*_2_ with the same symmetry but different values of the ratio *Q*_1_/*Q*_2_. At temperatures *T* in the range *T*_2_ < *T* < *T*_c_, the order parameter Q_1_ dominates over *Q*_2_, because the non-zero value of *Q*_2_ is induced via the coupling coefficient *D*, whereas for *T* < *T*_2_, the order parameter *Q*_2_ increases because of the change of sign of the coefficient *A*_2_. Hence, around *T*_2_, many macroscopic properties of the crystal (such as heat capacity and elastic constants) will show similar types of behaviour to those that would be expected to occur at a structural phase transition. Therefore, in the experimental context (and provided no further structural evidence is available), the ‘crossover’ described above could readily be confused with a phase transition, with or without symmetry breaking [51].

Now, the minimization of equation (3.5) gives

3.12 $$Q_{2}=-\frac{1}{D}(A_{1}Q_{1}+B_{1}^{\prime }{\left(Q_{1}\right)}^{3}+C_{1}{\left(Q_{1}\right)}^{5}).$$

Putting this expression for *Q*_2_ into equation (3.5) leads to a complicated even power expression of degree 15 (not given here) for Δ*F* in (*Q*_1_)^2^, the analytical treatment of which is unworkable in practice. Nevertheless, the true minimum of Δ*F* together with the equilibrium value of *Q*_1_ can be determined numerically. Then, the equilibrium value of *Q*_2_ is deduced from equation (3.12) and the excess heat capacity (Δ*C*_p_) can also be simulated numerically from the relation:

3.13 $$\Delta C_{p}=-T\frac{\partial^{2}\Delta F}{\partial T^{2}}.$$

To proceed further with numerical simulations, we have normalized the order parameters to unity (*Q*_1_ = *Q*_2_ = 1) at absolute zero temperature. In the absence of coupling (i.e. with *D* = 0), we obtain

3.14 $$T_{1}=\frac{{B^{\prime }}_{1}+C_{1}}{a_{1}}\;\text{and}\;T_{2}=\frac{B_{2}+C_{2}}{a_{2}}.$$

Furthermore, the jump (denoted as *Q*_1c_) in *Q*_1_ at *T*_c_ (corresponding to the first-order phase transition) is given by

3.15 $${\left(Q_{1c}\right)}^{2}=-\frac{3{B^{\prime }}_{1}}{4C_{1}}$$

with $B_{1}^{\prime }<0$ as specified above. Hence, we are left with seven independent free parameters, specifically: (*Q*_1c_)^2^, *T*_1_, *T*_2_, *C*_1_, *B*_2_, *C*_2_ and *D*.

## 4. Comparison with experimental data

We recall that the *n*-nonadecane/urea and *n*-hexadecane/urea inclusion compounds have been reported to undergo two successive phase transitions at *T*_c1_ (about 157 K on cooling for *n*-nonadecane/urea and about 150 K on cooling for *n*-hexadecane/urea) and at *T*_c2_ (about 140 K on cooling for *n*-nonadecane/urea and about 125 K on cooling for *n*-hexadecane/urea), corresponding to the phase sequence I → II → ‘III’ on decreasing temperature as discussed above [12,28,30].

In §3, we have proposed instead that the ‘transition’ at *T*_c2_ actually corresponds to a ‘crossover’ between two competing order parameters belonging to the same symmetry. Now, the observed phase transition at *T*_c1_ is clearly first order, given the abrupt changes in lattice parameters (and hence in the spontaneous strain *e*_1_–*e*_2_) and the thermal hysteresis observed at this transition [12,29,57], which implies that $B_{1}^{\prime }<0$ (and *C*_1_ > 0) in equation (3.5). We also recall that two zone boundary order parameters *Q*_1_ and *Q*_2_ both contribute to the new reflections (*h*, *k*, *l′*, *m′*) that appear in the low-temperature phase in positions with *h + k* odd, including main reflections for the host substructure (with *m′* = 0), main reflections for the guest substructure (with *l′* = 0) and satellite reflections (with *l′* ≠ 0 and *m′* ≠ 0). We emphasize that the respective contributions of *Q*_1_ and *Q*_2_ may be different for different *n*-alkane/urea inclusion compounds, and hence may explain differences in behaviour such as those observed [12,28] between *n*-nonadecane/urea and *n*-hexadecane/urea.

**Figure 5.**
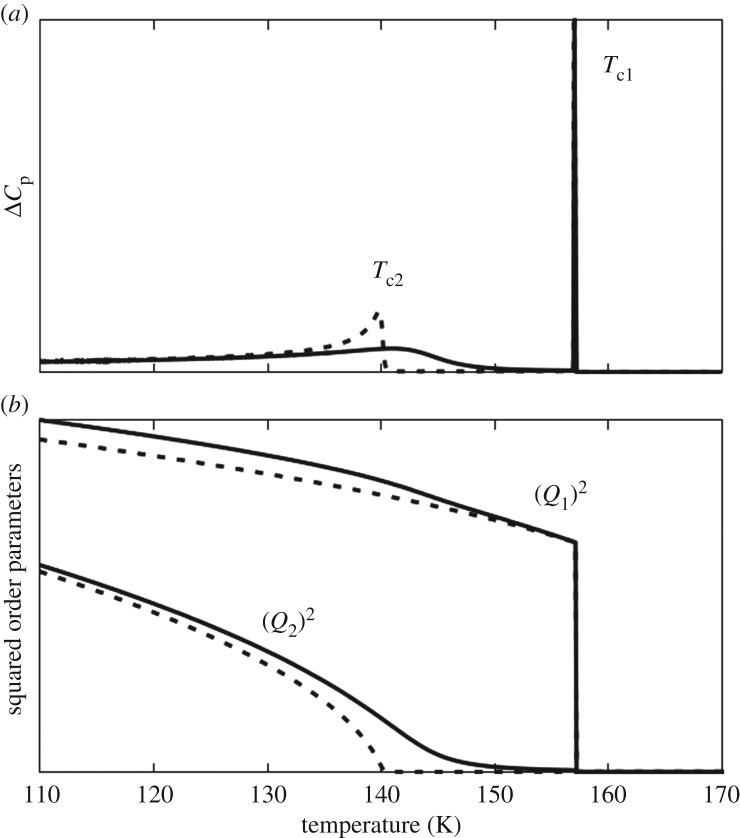
Calculations of experimental data as a function of temperature for *n-*nonadecane/urea using our phenomenological model: (*a*) excess heat capacity at constant pressure and (*b*) squared order parameters. The data were calculated (see equations (3.5), (3.12) and (3.13)) for *T*_c1_ = 157 K and *T*_c2_ = 140 K (corresponding to the reported phase transition temperatures for *n*-nonadecane/urea on cooling) together with the values of the parameters discussed in the text and for two different values of the coupling parameter: *D* = 10^−4^ (dashed lines) and *D* = 5 × 10^−3^ (continuous lines).

We now compare our model potential with experimental data for *n*-nonadecane/urea, using the following strategy to select suitable values of the parameters [(*Q*_1c_)^2^, *T*_1_, *T*_2_, *C*_1_, *B*_2_, *C*_2_ and *D*] in our model. First, the jump of (*Q*_1_)^2^ at *T*_c1_ [denoted as (*Q*_1c_)^2^] was fixed at 0.5 in order to comply qualitatively with the experimental results obtained for *e*_s_ = (*e*_1_–*e*_2_) at *T*_c1_ for *n*-nonadecane/urea [57] and *n*-hexadecane/urea [12] (see equations (3.4) and (3.15)). Then, the value of *T*_1_ was set to 126 K, corresponding to *T*_c1_ ≈ 157 K (as observed for *n*-nonadecane/urea), and the value of *T*_2_ was set to 140 K, corresponding to *T*_c2_ ≈ 140 K (as observed for *n*-nonadecane/urea). The coefficients *B*_2_, *C*_1_ and *C*_2_ were adjusted manually so that the thermal anomaly around *T*_c2_ is sufficiently narrow and intense. Finally, we have found that very weak values of the coupling coefficient *D* are required in order to reproduce the experimental data qualitatively (this observation is completely consistent with previous studies [47,58], which concluded that the reorientational and translational motions of the *n*-alkane guest molecules in *n*-alkane/urea inclusion compounds are uncorrelated). The results of our calculations using *C*_1_ = 0.5, *B*_2_ = 0.1 and *C*_2_ = 0.9, together with either *D* = 10^−4^ or *D* = 5 × 10^−3^, are shown in figure 5.

Clearly, our calculated data for the excess heat capacity Δ*C*_p_ as a function of temperature (figure 5*b*) are in good qualitative agreement with experimental DSC data reported for *n*-nonadecane/urea [29] and *n*-hexadecane/urea [12]. Thus, a strong thermal anomaly is associated with the first-order transition at *T*_c1_, and only a weak thermal anomaly is associated with the ‘crossover’ at *T*_c2_.

**Figure 6.**
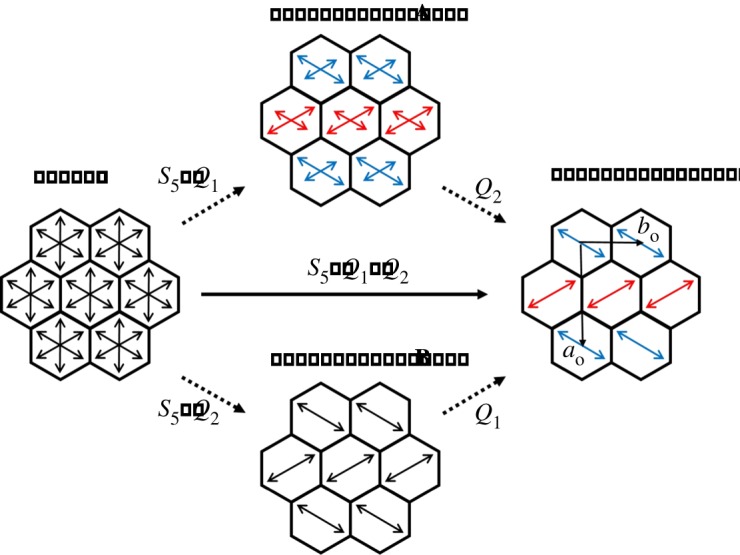
Schematic representation, viewed along the tunnel axis (**c**-axis), of the orientations of the *n*-alkane guest molecules in phases I and II of the *n*-nonadecane/urea and *n*-hexadecane/urea inclusion compounds. The orientations of the guest molecules, projected onto the *ab-*plane, are shown as arrows. In phase I, the guest molecules are equally distributed among six energetically equivalent orientations. In the initial state of phase II for *n*-nonadecane/urea (state **A**), herringbone ordering of the guest molecules is partially achieved, with the lengths of the arrows symbolizing the relative probability of different orientations. Guest molecules displaced by +*δ*_g_/2 along the tunnel axis are shown as blue arrows and guest molecules displaced by −*δ*_g_/2 are shown as red arrows. In the initial state of phase II for *n*-hexadecane/urea (state **B**), complete ‘herringbone’ ordering of the guest molecules is achieved. In the final state of phase II for both *n*-nonadecane/urea and *n*-hexadecane/urea, herringbone ordering is achieved and the guest molecules adopt the same displacements along the tunnel axis as in state **A**.

The temperature dependences of (*Q*_1_)^2^ and (*Q*_2_)^2^, calculated using the phenomenological parameters specified above, are shown in figure 5*b*. According to the relations in equation (3.4), (*Q*_1_)^2^ is proportional to both *S*_5_ and *e*_s_. No direct measurement of *S*_5_ has been reported so far, but *e*_s_ can be determined from the lattice parameters *a*_o_ and *b*_o_ (equation (3.3)) for *n*-nonadecane/urea [57] and *n*-hexadecane/urea [12]. On qualitative grounds, the results shown in figure 5*b* agree with experimental results ([57, fig. I-8] and [12, fig. 6]), which exhibit a marked jump of *e*_s_ at the temperature *T*_c1_ of the first-order phase transition, followed by a damped regular increase at lower temperatures, as expected for a ‘weak’ first-order phase transition.

As mentioned above, *Q*_1_ and *Q*_2_ are related to the intensities of the main reflections with *h *+ *k* odd and the intensities of the satellite reflections with *h *+ *k* odd in the low-temperature phase. Furthermore, the intensities of these peaks are proportional to the square of the order parameter responsible for the phase transition [59]. Thus, plots of (*Q*_1_)^2^ versus *T* and (*Q*_2_)^2^ versus *T* calculated using our model (figure 5*b*) can be compared directly to the intensities of the relevant reflections as a function of temperature.

In the case of *n*-nonadecane/urea containing fully deuterated guest molecules, an abrupt increase in the intensities of the satellite reflections (*h*, *k*, *l′*, *m′*) with *l′* ≠ 0, *m′* ≠ 0 and *h + k* odd has been reported [28, fig. 3b] at the transition near *T*_c1_, followed by a regular increase up to a maximum and then a slow decrease down to about *T*_c2_. This reported behaviour is consistent with the results obtained from our ad hoc model, except for the existence of the maximum between *T*_c1_ and *T*_c2_. At the same time, the main reflections of the host substructure (*h*, *k*, *l′*, 0) with *h + k* odd and the main reflections of the guest substructure (*h*, *k*, 0, *m′*) with *h + k* odd are very weak, but it is evident [28, fig. 3b] that their intensities increase as temperature decreases, which is the predicted behaviour for (*Q*_2_)^2^ according to our model (figure 5*b*) and is in good agreement with experimental observations that we reported recently [31]. Below 130 K, the intensities of these reflections exhibit a marked and progressive increase [28, fig. 3], as expected from our model (figure 5*b*).

For *n*-hexadecane/urea, it is reported [12] that the main reflections with *h + k* odd first appear abruptly at *T*_c1_ ≈ 150 K, whereas the intensities of the satellite reflections with *h + k* odd grow progressively at temperatures below about 130 K [12, fig. 11]. Significantly, these observations are also in qualitative agreement with our model (figure 5*b*), but with the roles of *Q*_1_ and *Q*_2_ reversed compared to the situation discussed above for *n*-nonadecane/urea. Thus, for *n*-hexadecane/urea, *Q*_2_ must be considered as the driving order parameter instead of *Q*_1_ when the coupling occurs as (*Q*_2_)^2^*S*_5_ and (*Q*_2_)^2^*e*_s_ rather than as (*Q*_1_)^2^*S*_5_ and (*Q*_1_)^2^*e*_s_.

Our analysis demonstrates that two different contributions govern the intensities of the main reflections with *h + k* odd and the intensities of the satellite reflections with *h + k* odd, in relation to the two-order parameters *Q*_1_ and *Q*_2_. Hence, we propose that the intensity of the satellite reflections is mainly associated with the $T_{z}^{\prime }$ zone boundary sliding mode polarized along the **c**-axis (*Q*_1_), as it is related to the *incommensurate* properties of the composite crystal along the tunnel axis, and we propose that the intensity of the main reflections is essentially governed by the pseudo-spin coordinate $S_{6}^{\prime }$ (*Q*_2_), as it is responsible for the *commensurate* ‘herringbone’ ordering of the *n*-alkane guest molecules in the *a*_o_*b*_o_-plane. Thus, for *n*-nonadecane/urea, it turns out that the zone boundary sliding mode $T_{z}^{\prime }$ (*Q*_1_) first condenses at *T*_c1_ together with the pseudo-spin coordinate *S*_5_, creating the spontaneous strain *e*_s_, while the pseudo-spin coordinate $S_{6}^{\prime }$ (*Q*_2_) is almost inactive. As a result, orientational ordering of the *n*-nonadecane guest molecules is partially achieved due to *S*_5_ and a small contribution of $S_{6}^{\prime }$ (i.e. *Q*_1_ >> *Q*_2_), whereas the anti-translations of the *n*-nonadecane guest molecules along the tunnel direction have already condensed, resulting in the intermediate ‘state **A**’ of phase II schematized in figure 6. Below the ‘crossover’ temperature *T*_2_, $S_{6}^{\prime }$ (*Q*_2_) then condenses to achieve the final state of phase II with a complete ‘herringbone’ orientational ordering of the *n*-nonadecane guest molecules.

In the case of *n*-hexadecane/urea, for which the roles of *Q*_1_ and *Q*_2_ are reversed, the pseudo-spin coordinate $S_{6}^{\prime }$ (*Q*_2_) first condenses at *T*_c1_ together with S_5_, thus achieving complete ‘herringbone’ ordering of the *n*-hexadecane guest molecules, while $T_{z}^{\prime }$ (*Q*_1_) is almost inactive (i.e. *Q*_2 _>> *Q*_1_), resulting in the intermediate ‘state **B**’ of phase II schematized in figure 6. Then, below the ‘crossover’ temperature (*T*_1_ in this case), the zone boundary sliding mode $T_{z}^{\prime }$ (*Q*_1_) condenses to give the final state of phase II (figure 6).

Clearly, the difference between the *n*-nonadecane/urea and *n*-hexadecane/urea inclusion compounds may be interpreted in terms of differences in the coupling forces between *Q*_1_, *Q*_2_, *S*_5_ and *e*_s_.

## 5. Concluding remarks

Based on a wide range of published data relating to structural phase transitions in the incommensurate *n*-nonadecane/urea and *n*-hexadecane/urea inclusion compounds, and based on symmetry considerations, we have identified the order parameters necessary to explain the I ↔ II ↔ ‘III’ phase sequence reported recently in the literature for these materials. Specifically, the order parameters are zone centre and zone boundary pseudo-spin coordinates, the coupling of which accounts for ‘herringbone’ orientational ordering of the *n*-alkane guest molecules and explains the transition from the orientationally disordered hexagonal *P*6_1_22(00*γ*) phase I to the orientationally ordered orthorhombic *P*2_1_2_1_2_1_(00*γ*) phase II. Also involved are the macroscopic strain (*e*_1_–*e*_2_), which is responsible for the orthorhombic distortion, and the so-called *zone boundary sliding mode* of the guest substructure relative to the host substructure.

We have elaborated a simplified thermodynamic Landau potential taking into account the fact that phases II and ‘III’ belong to the same (3 + 1)-dimensional superspace group *P*2_1_2_1_2_1_(00*γ*). This model can account qualitatively for the temperature dependence of the spontaneous strain and the temperature dependence of the intensities of main reflections and satellite reflections. Hence, we have shown that the reported II ↔ ‘III’ phase transition should actually be regarded as a ‘crossover’ between two competing order parameters with the same symmetry: specifically, the zone boundary pseudo-spin coordinate and the zone boundary sliding mode. So, we conclude that the herringbone orientational ordering and the translational freezing of the *n*-alkane guest molecules take place in two steps within a single phase II, with these two steps occurring in the reverse order on decreasing temperature for *n*-nonadecane/urea and *n*-hexadecane/urea. Furthermore, our results show conclusively that the physical basis of the phase transition mechanisms in these incommensurate composite materials does not require the introduction of a higher-dimensional [(3 + 2)-dimensional] description [30] in superspace.

Finally, in the context of the (3 + 1)-dimensional description of the superspace group in phase II of *n*-nonadecane/urea, the fact that satellite reflections are indexed with large values of *l′* and *m′* [31] may appear unusual. However, we note that examples of incommensurate systems with high values of indices for satellite reflections arise when the modulation function becomes discontinuous and a non-analytical ‘soliton regime’ can exist [60].

## Data accessibility

This article has no additional data.

## References

[1] Hollingsworth MD, Harris KDM 1996 Urea, thiourea, and selenourea. In Comprehensive supramolecular chemistry (eds Atwood JL, Davies JED, MacNicol DD, Vögtle F), pp. 177–237. Oxford, UK: Pergamon Press.

[2] Rennie AJO, Harris KDM 1990 A mathematical model of one-dimensional inclusion compounds: a new approach towards understanding commensurate and incommensurate behaviour. Proc. R. Soc. Lond. A 430, 615–640. (doi:10.1098/rspa.1990.0109)

[3] Harris KDM 1993 Investigating the structure and dynamics of a family of organic solids: the alkane/urea inclusion compounds. J. Solid State Chem. 106, 83–98. (doi:10.1006/jssc.1993.1267)

[4] van Smaalen S, Harris KDM 1996 Superspace group descriptions of the symmetries of incommensurate urea inclusion compounds. Proc. R. Soc. Lond. A 452, 677–700. (doi:10.1098/rspa.1996.0034)

[5] Weber T, Boysen H, Honal M, Frey F, Neder RB 1996 Diffuse and satellite scattering in urea inclusion compounds with various alkane molecules. Z. Kristallogr. 211, 238–246. (doi:10.1524/zkri.1996.211.4.238)

[6] Lefort R, Etrillard J, Toudic B, Guillaume F, Breczewski T, Bourges P 1996 Incommensurate intermodulation of an organic intergrowth compound observed by neutron scattering. Phys. Rev. Lett. 77, 4027–4030. (doi:10.1103/PhysRevLett.77.4027) 10062369 10.1103/PhysRevLett.77.4027

[7] Smith AE 1952 The crystal structure of the urea–hydrocarbon complexes. Acta Crystallogr. 5, 224–235. (doi:10.1107/S0365110X52000629)

[8] Harris KDM, Thomas JM 1990 Structural aspects of urea inclusion compounds and their investigation by X-ray diffraction: a general discussion. J. Chem. Soc. Faraday Trans. 86, 2985–2996. (doi:10.1039/FT9908602985)

[9] Rennie AJO, Harris KDM 1992 Is the guest periodicity of CH_3_(CH_2_)_*n*_CH_3_/urea inclusion compounds linearly dependent on *n*? A mathematical analysis. Chem. Phys. Lett. 188, 1–4. (doi:10.1016/0009-2614(92)85078-O)

[10] Rennie AJO, Harris KDM 1992 A quantitative analysis of guest periodicity in one-dimensional inclusion compounds. J. Chem. Phys. 96, 7117–7124. (doi:10.1063/1.462545)

[11] Shannon IJ, Harris KDM, Rennie AJO, Webster MB 1993 Theoretical prediction of the guest periodicity of alkane/urea inclusion compounds. J. Chem. Soc. Faraday Trans. 89, 2023–2029. (doi:10.1039/FT9938902023)

[12] Huard M, Toudic B, Rabiller P, Ecolivet C, Guérin L, Bourges P, Breczewski T, Hollingsworth MD 2011 Confined linear molecules inside an aperiodic supramolecular crystal: the sequence of superspace phases in *n*-hexadecane/urea. J. Chem. Phys. 135, 204505 (doi:10.1063/1.3663711) 22128941 10.1063/1.3663711

[13] Fukao K 1990 Disorder in paraffin chains of urea adducts and *n*-paraffins. J. Chem. Phys. 92, 6867–6874. (doi:10.1063/1.458274)

[14] Le Lann H, Odin C, Toudic B, Ameline JC, Gallier J, Guillaume F, Breczewski T 2000 Single-crystal deuterium NMR study of the symmetry breaking in an incommensurate organic inclusion compound. Phys. Rev. B 62, 5442–5451. (doi:10.1103/PhysRevB.62.5442)

[15] Rabiller P, Etrillard J, Toupet L, Kiat JM, Launois P, Petricek V, Breczewski T 2001 Disorder versus structure analysis in intergrowth urea inclusion compounds. J. Phys. Condens. Matter 13, 1653–1668. (doi:10.1088/0953-8984/13/8/304)

[16] Chatani Y, Taki Y, Tadokoro H 1977 Low-temperature form of urea adducts with *n*-paraffins. Acta Crystallogr. Sect. B 33, 309–311. (doi:10.1107/S0567740877003501)

[17] Forst R, Boysen H, Frey F, Jagodzinski H, Zeyen C 1986 Phase transitions and ordering in urea inclusion compounds with *n*-paraffins. J. Phys. Chem. Solids 47, 1089–1097. (doi:10.1016/0022-3697(86)90077-6)

[18] Forst R, Jagodzinski H, Boysen H, Frey F 1987 Diffuse scattering and disorder in urea inclusion compounds OC(NH_2_)_2_+C_*n*_H_2*n*+2_. Acta Crystallogr. Sect. B 43, 187–197. (doi:10.1107/S0108768187098082)

[19] Forst R, Jagodzinski H, Boysen H, Frey F 1990 The disordered crystal structure of urea inclusion compounds OC(NH_2_)_2_ + C_*n*_H_2*n*+2_. Acta Crystallogr. Sect. B 46, 70–78. (doi:10.1107/S0108768189009912)

[20] Fukao K, Horiuchi T, Taki S, Matsushige K 1990 Phase transitions of urea adducts with *n*-paraffins under high pressure. Mol. Cryst. Liq. Cryst. 180, 405–416. (doi:10.1080/00268949008042221)

[21] Harris KDM, Gameson I, Thomas JM 1990 Powder X-ray diffraction studies of a low-temperature phase transition in the *n*-hexadecane/urea inclusion compound. J. Chem. Soc. Faraday Trans. 86, 3135–3143. (doi:10.1039/FT9908603135)

[22] Lynden-Bell RM 1993 The orientational order/disorder phase transition of urea–paraffin inclusion compounds. Mol. Phys. 79, 313–321. (doi:10.1080/00268979300101231)

[23] Schmicker D, van Smaalen S, de Boer JL, Haas C, Harris KDM 1995 Observation of the sliding mode in incommensurate intergrowth compounds: Brillouin scattering from the inclusion compound of urea and heptadecane. Phys. Rev. Lett. 74, 734–737. (doi:10.1103/PhysRevLett.74.734) 10058834 10.1103/PhysRevLett.74.734

[24] Yeo L, Kariuki BM, Serrano-González H, Harris KDM 1997 Structural properties of the low-temperature phase of the hexadecane/urea inclusion compound, investigated by synchrotron X-ray powder diffraction. J. Phys. Chem. B 101, 9926–9931. (doi:10.1021/jp971607d)

[25] Ollivier J, Ecolivet C, Beaufils S, Guillaume F, Breczewski T 1998 Light scattering by low-frequency excitations in quasi-periodic *n*-alkane/urea adducts. Europhys. Lett. 43, 546–551. (doi:10.1209/epl/i1998-00395-x)

[26] Pemberton RC, Parsonage NG 1965 Thermodynamic properties of urea + hydrocarbon adducts. Part 1. Heat capacities of the adducts of *n*-C_10_H_22_, n-C_12_H_26_, n-C_16_CH_34_ and n-C_20_H_42_ from 12 to 300 K. Trans. Faraday Soc. 61, 2112–2121. (doi:10.1039/TF9656102112)

[27] Pemberton RC, Parsonage NG 1966 Thermodynamic properties of urea-hydrocarbon adducts. Part 2. Heat capacities of adducts of *n*-pentadecane and 2-methyl-pentadecane from 12 to 300 K. Trans. Faraday Soc. 62, 553–557. (doi:10.1039/TF9666200553)

[28] Toudic B et al. 2008 Hidden degrees of freedom in aperiodic materials. Science 319, 69–71. (doi:10.1126/science.1146745) 18174435 10.1126/science.1146745

[29] López-Echarri A, Ruiz-Larrea I, Fraile-Rodríguez A, Díaz-Hernández J, Breczewski T, Bocanegra EH 2007 Phase transitions in the urea/*n*-nonadecane system by calorimetric techniques. J. Phys. Condens. Matter 19, 186221 (doi:10.1088/0953-8984/19/18/186221) 21691002 10.1088/0953-8984/19/18/186221

[30] Toudic B, Rabiller P, Bourgeois L, Huard M, Ecolivet C, McIntyre GJ, Bourges P, Breczewski T, Janssen T 2011 Temperature–pressure phase diagram of an aperiodic host guest compound. Europhys. Lett. 93, 16003 (doi:10.1209/0295-5075/93/16003)

[31] Couzi M, Guillaume F, Harris KDM, Palmer BA, Christensen K, Collins SP 2016 The true structural periodicities and superspace group descriptions of the prototypical incommensurate composite materials: alkane/urea inclusion compounds. Europhys. Lett. 116, 56001 (doi:10.1209/0295-5075/116/56001)

[32] Mariette C et al. 2013 Critical phenomena in higher dimensional spaces: the hexagonal-to-orthorhombic phase transition in aperiodic *n*-nonadecane/urea. Phys. Rev. B 87, 104101 (doi:10.1103/PhysRevB.87.104101)

[33] Zerdane S, Mariette C, McIntyre GJ, Lemée-Cailleau M-H, Rabiller P, Guérin L, Ameline JC, Toudic B 2015 Neutron Laue and X-ray diffraction study of a new crystallographic superspace phase in *n*-nonadecane–urea. Acta Crystallogr. Sect. B 71, 293–299. (doi:10.1107/S2052520615005442) 10.1107/S2052520615005442 26027005

[34] Toudic B, Guérin L, Mariette C, Frantsuzov I, Rabiller P, Ecolivet C, Janssen T, Hollingsworth MD 2017 Comment on ‘The true structural periodicities and superspace goup descriptions of the prototypical incommensurate composite materials: alkane/urea inclusion compounds’. Europhys. Lett. 119, 660004 (doi: 10.1209/0295-5075/119/66004)

[35] Marti-Rujas J, Desmedt A, Harris KDM, Guillaume F 2004 Direct time-resolved and spatially resolved monitoring of molecular transport in a crystalline nanochannel system. J. Am. Chem. Soc. 126, 11 124–11 125. (doi:10.1021/ja040117d) 10.1021/ja040117d 15355073

[36] Martí-Rujas J, Harris KDM, Desmedt A, Guillaume F 2006 Significant conformational changes associated with molecular transport in a crystalline solid. J. Phys. Chem. B 110, 10 708–10 713. (doi:10.1021/jp060738o) 10.1021/jp060738o 16771317

[37] Martí-Rujas J, Desmedt A, Harris KDM, Guillaume F 2007 Kinetics of molecular transport in a nanoporous crystal studied by confocal Raman microspectrometry: single-file diffusion in a densely filled tunnel. J. Phys. Chem. B 111, 12 339–12 344. (doi:10.1021/jp076532k) 10.1021/jp076532k 17924692

[38] Martí-Rujas J, Desmedt A, Harris KDM, Guillaume F 2009 Bidirectional transport of guest molecules through the nanoporous tunnel structure of a solid inclusion compound. J. Phys. Chem. C 113, 736–743. (doi:10.1021/jp806380p)

[39] Mariette C, Guérin L, Rabiller P, Chen Y-S, Bosak A, Popov A, Hollingsworth MD, Toudic B 2014 The creation of modulated monoclinic aperiodic composites in *n*-alkane/urea compounds. Z. Kristallogr. 230, 5–11. (doi:10.1515/zkri-2014-1773) 10.1515/zkri-2014-1773 PMC4512510 26213678

[40] Bradley CJ, Cracknell AP 1972 The mathematical theory of symmetry in solids: representation theory for point groups and space groups. Oxford, UK: Clarendon Press.

[41] Stokes HT, Hatch DM. 1988 Isotropy subgroups of the 230 crystallographic space groups. Singapore: World Scientific.

[42] Toledano J-C, Toledano P. 1987 The Landau theory of phase transitions. Singapore: World Scientific.

[43] Guillaume F, Sourisseau C, Dianoux AJ 1990 Inelastic incoherent neutron scattering study of molecular motions of *n*-nonadecane in urea clathrate. J. Chem. Phys. 93, 3536–3541. (doi:10.1063/1.458835)

[44] Guillaume F, Sourisseau C, Dianoux A 1991 Rotational and translational motions of *n*-nonadecane in the urea inclusion compound as evidenced by incoherent quasi-elastic neutron-scattering. J. Chim. Phys.-Chim. Biol. 88, 1721–1739. (doi:10.1051/jcp/1991881721)

[45] Souaille M, Guillaume F, Smith JC 1996 Molecular dynamics simulation of *n*-nonadecane in urea inclusion compound. I. Comparison with quasielastic neutron scattering experiment. J. Chem. Phys. 105, 1516–1528. (doi:10.1063/1.472030)

[46] Souaille M, Guillaume F, Smith JC 1996 Molecular dynamics simulation of *n*-nonadecane in urea inclusion compound. II. Rotational distribution and elastic incoherent structure factor. J. Chem. Phys. 105, 1529–1536. (doi:10.1063/1.472013)

[47] Souaille M, Smith JC, Guillaume F 1997 Simulation of collective dynamics of *n*-nonadecane in the urea inclusion compound. J. Phys. Chem. B 101, 6753–6757. (doi:10.1021/jp9705885)

[48] Desmedt A, Kitchin SJ, Guillaume F, Couzi M, Harris KDM, Bocanegra EH 2001 Phase transitions and molecular dynamics in the cyclohexane/thiourea inclusion compound. Phys. Rev. B 64, 054106 (doi:10.1103/PhysRevB.64.054106)

[49] Etrillard J, Lasjaunias JC, Toudic B, Guillaume F, Breczewski T 2000 Low-frequency dynamics in molecular incommensurate composite: specific heat of nonadecane/urea inclusion compound. Europhys. Lett. 49, 610–616. (doi:10.1209/epl/i2000-00194-y)

[50] Nye JF 1957 Physical properties of crystals: their representation by tensors and matrices. Oxford, UK: Oxford University Press.

[51] Salje EKH 1990 Phase transitions in ferroelastic and co-elastic crystals. Cambridge, UK: Cambridge University Press.

[52] Toudic B, Lefort R, Ecolivet C, Guérin L, Currat R, Bourges P, Breczewski T 2011 Mixed acoustic phonons and phase modes in an aperiodic composite crystal. Phys. Rev. Lett. 107, 205502 (doi:10.1103/PhysRevLett.107.205502) 22181743 10.1103/PhysRevLett.107.205502

[53] Breczewski T, López-Echarri A, Rubio-Peña L, Aroyo MI, Ruiz-Larrea I, Bocanegra EH 2007 Experimental study of the ferroelastic phase transition in urea/*n*-heptadecane composite. J. Phys. Chem. B 111, 5218–5224. (doi:10.1021/jp067115v) 17451266 10.1021/jp067115v

[54] Salje E 1985 Thermodynamics of sodium feldspar I: order parameter treatment and strain induced coupling effects. Phys. Chem. Miner. 12, 93–98. (doi:10.1007/BF01046833)

[55] Salje E, Kuscholke B, Wruck B, Kroll H 1985 Thermodynamics of sodium feldspar II: experimental results and numerical calculations. Phys. Chem. Mine. 12, 99–107. (doi:10.1007/BF01046834)

[56] Salje E, Devarajan V 1986 Phase transitions in systems with strain-induced coupling between two order parameters. Phase Transitions 6, 235–247. (doi:10.1080/01411598608218308)

[57] Lefort R 1998 Effets structuraux et dynamiques dans les cristaux quasipériodiques composites d'urée/alcane. PhD thesis, Université de Rennes 1, France.

[58] Guillaume F 1994 Helical motions of aliphatic chains in molecular crystals the incoherent quasi-elastic neutron scattering law. Mol. Phys. 81, 1411–1423. (doi:10.1080/00268979400100961)

[59] Cowley RA 1980 Structural phase transitions I. Landau theory. Adv. Phys. 29, 1–110. (doi:10.1080/00018738000101346)

[60] Pérez-Mato JM, Madariaga G 1986 X-ray diffraction of incommensurate structures in the soliton regime. Solid State Commun. 58, 105–109. (doi: 10.1016/0038-1098(86)90864-1)

